# Translation, Cross-Cultural Adaptation and Validation of the Lymphedema Quality of Life Questionnaire (LYMQOL) in German-Speaking Patients with Lymphedema of the Lower Limbs

**DOI:** 10.3390/healthcare12030409

**Published:** 2024-02-05

**Authors:** Rima Nuwayhid, Mary Lee Warg, Simon Heister, Stefan Langer, Torsten Schulz

**Affiliations:** Department of Orthopaedic, Trauma and Plastic Surgery, University Hospital Leipzig, 04103 Leipzig, Germanysimon.heister@medizin.uni-leipzig.de (S.H.); torsten.schulz@medizin.uni-leipzig.de (T.S.)

**Keywords:** lymphedema, LYMQOL, German, lower limbs, quality of life, cross-cultural adaptation

## Abstract

The LYMQOL Leg questionnaire is the most widely used, evidence-based tool for the assessment of health-related quality of life (HRQoL) in patients with lower limb lymphedema (LLL). It has been translated into several languages, but a German version is currently lacking. The aim of our study was to validate a German translation of LYMQOL Leg. Translation and cross-cultural adaptation were performed in accordance with ISPOR principles. A total of 103 patients with LLL from Germany, Austria, and Switzerland were interviewed twice. The content and face validity assessments indicated that the German LYMQOL Leg questionnaire was acceptable for interviewing patients with lymphedema. Comparing the LYMQOL Leg with the SF-36 demonstrated good construct validity. Reliability determined by the test–retest procedure was good (intra-class-correlation coefficients 0.68–0.92). Cronbach’s alpha values ranged from 0.76 to 0.90 in both interviews, showing an acceptable internal consistency. The four domains of the questionnaire reached a cumulative variance of 52.7% in the factor analysis. The association between the lymphedema stages and the LYMQOL Leg domain scores was not significant. In conclusion, the validity of the German version of LYMQOL Leg, called *LYMQOL Bein*, was confirmed and thus represents a suitable tool for measuring HRQoL in German-speaking patients with LLL.

## 1. Introduction

Lymphedema is the result of an impaired lymphatic system leading to an interstitial accumulation of protein-rich fluid and clinically presenting as swelling of the affected region, mostly the limbs [1]. Globally, the most common cause of secondary lymphedema is the parasitic disease filariasis, affecting 90 million people [2]. In contrast, in developed countries, most lymphedema is the adverse effect of cancer and its treatment [2,3]. Only an estimated 1% of lymphedemas are considered primary [3]. Lymphedema patients are at risk of developing lymphangiosarcoma as a rare but fatal complication of this chronic and progressive disease [4].

Reporting on health-related quality of life (HRQoL) in lymphedema patients mainly focuses on breast cancer survivors with upper limb lymphedema (ULL) [5]. Nevertheless, there is ample evidence for the negative impact of lower limb lymphedema (LLL) on functional and psychosocial well-being, daily activities, pain, and global QoL [5,6,7,8]. Patient-centred care puts emphasis on the patient’s perspective on their own symptoms, well-being, and assessment of therapies. Patient-reported outcome measures (PROMs) are tools to record and measure health or aspects of it coming directly from the patient without a therapist’s interference [9]. In particular, quality of life should be assessed by patients themselves to avoid observer bias [10]. Interestingly, lymphedema severity rated by the International Society of Lymphology (ISL) stage does not seem to correlate with lower HRQoL, which underlines the need for lymphedema-specific PROM [11].

A validated German questionnaire on HRQoL in patients with chronic venous disease, the FLQA-V, was redesigned and validated to the needs of lymphedema patients and published in 2005 as the *Freiburg Life Quality Assessment for Lymphedema*, FLQA-L [12]. As the original FLQA-L consisted of 92 items, a validated short form with 33 items was introduced in 2018 as FLQA-LS [13]. Still, it is not commonly used; thus, studies in German-speaking cohorts resort to using tools, which have not been validated in this specific population. One study used a translated but not validated version of the Lymphedema Quality of Life Inventory (LyQLI) by Klernäs et al., which, in its abbreviated version, still consists of 45 items [14,15]. Another study used a questionnaire intended for patients with chronic venous insufficiency [16,17]. The German EORTC QLQ-C30, although aimed at cancer patients, was used in patients with primary and non-cancer-related secondary lymphedema [18]. Other researchers used general PROM not specifically targeted at lymphedema patients or developed their own questionnaires and put them to use without prior validation [19,20].

Recently, a validated German version of the LYMPH-Q upper extremity module has been published, which as its original is aimed exclusively at patients with upper limb lymphedema [21]. While a German version of Lymph-ICF does exist, the process of translation and validation was designed poorly, with the number of analysed interviews as low as 6 [22]. It, thus, has to be stated that no sound evidence for its translation and cultural adaptation was presented [22,23]. A recent systematic review found 19 PROMs intended to measure HQRoL in lymphedema patients and evaluated them based on criteria specified in the COSMIN (consensus-based standards for the selection of health measurement instruments) framework [24]. Out of these 19 PROMs, 7 were rated “sufficient for content validity”, with LYMQOL Arm and LYMQOL Leg among them [24]. Furthermore, LYMQOL Leg was rated “sufficient for structural validity, internal consistency, and hypothesis testing” and is the only PROM with an evidence-based recommendation [24]. 

LYMQOL was introduced in 2010 by Keeley et al. as a PROM for HQRoL in patients with ULL (LYMQOL Arm) or LLL (LYMQOL Leg) in clinical and scientific setups [25]. LYMQOL has since been used to evaluate HQRoL in lymphedema patients after different surgical and conservative treatments and even in children [26,27,28]. It is the most commonly used tool to assess health-related quality of life in patients with LLL [8,29]. The international demand for a facile tool has led to translations and cultural adaptations of the original English LYMQOL into several other languages. LYMQOL Leg has been translated into Korean, Turkish, Swedish, Chinese, Dutch, and Portuguese [30,31,32,33,34,35,36]. LYMQOL Arm has been translated into Korean, Turkish, Swedish, Chinese, and Italian and mostly validated in breast cancer patients [33,34,37,38,39,40]. Surprisingly, despite the need for a German evidence-based PROM on HQRoL in patients with LLL, it is still lacking [23].

The specific objective of this study was to provide a German translation and cross-cultural adaptation in accordance with ISPOR Principles of Good Practice for the Translation and Cultural Adaptation Process for PRO Measures of the LMYQOL Leg, as it is the PROM most commonly used and backed by most evidence.

## 2. Materials and Methods

### 2.1. LYMQOL Leg

The LYMQOL Leg is a condition-specific PROM consisting of 27 items to assess HQRoL in patients with LLL [25]. Questions are grouped into four domains of HQRoL: function (8 items), appearance and body image (7 items), symptoms (5 items), and mood (6 items). These items are rated on a Likert-like scale with four grades ranging from “not at all” (1) to “a lot” (4). Each domain is evaluated individually by adding up the scores (1 to 4) and dividing this sum by the number of items in the respective domain. Accordingly, a low LYMQOL score indicates higher HQRoL (unlike SF-36, see below). Unanswered or not applicable questions are scored with 0, and if half or more questions in any domain are not answered, this domain may not be evaluated. The function domain is supplemented by a text field asking for examples of effects on leisure activities. Finally, global quality of life is indicated on a numeric rating scale from poor (0) to excellent (10). Keeley et al.’s original publication contains two versions of LYMQOL Leg. After further statistical analysis of the answers given in the process of validation, internal correlation using Spearman’s correlation coefficient (rho), items were deemed redundant and removed from the thus shorter final version. We followed these alterations, with the exception of the question aimed at “relationship with your partner” (see discussion). Furthermore, we included the question of subjectively perceived severity of the swelling of the affected limb using the same grading scale as in the preceding questions, as proposed by Wedin et al. in their Swedish version of the LYMQOL [33]. This was modified to also clearly indicate which leg is affected, with both legs being a possible answer.

### 2.2. Short-Form Health Survey (SF-36)

To assess construct and criterion validity, we chose the SF-36 for comparison. The SF-36 is a multidimensional, generic questionnaire that is commonly used to evaluate global HRQoL by PROM [41,42]. A German version has been validated [43]. With a total of 36 questions subgrouped into eight domains, they are summarised as physical component summary (PCS) and mental component summary (MCS). The MCS and PCS scores are calculated by adding weighted subscale scores for the German population with results ranging from 0 to 100 [44]. In contrast to LYMQOL, a low SF-36 score indicates lower HRQoL.

### 2.3. Translation Process

The translation process was planned and executed based on the ISPOR Principles of Good Practice for the Translation and Cultural Adaptation Process for Patient-Reported Outcomes (PRO) Measures. The first author of this publication was assigned project manager.

#### 2.3.1. Forward Translation

The original English publication of LYMQOL by Keeley et al. was handed out to six physicians and surgeons (three female and three male) who are native speakers of German and regularly treat lymphedema patients. Among them were two bilingual professionals (English and German). Beforehand, the purpose of this study and the LYMQOL itself were explained. It was stated that comprehensibility should be prioritised. Simultaneously, a professional translation and interpretation agency was mandated to translate the English LYMQOL to German by a native German speaker (female).

#### 2.3.2. Reconciliation

The resulting seven German versions were then compared by project manager and senior author, agreeing on one preliminary German version. 

#### 2.3.3. Back Translation

This preliminary German version was then sent to the translation and interpretation agency for a backwards translation into English by a native English speaker (male), who was not familiar with the original questionnaire.

#### 2.3.4. Back Translation Review and Harmonisation

The project manager and the initial professional translator held a harmonisation meeting to compare the back translation with the original by Keeley et al. Also, the preliminary German version was presented to the professional translator. Ascertaining the equivalence of our translation with the original, we reached a consensus on the prefinal German version of LYMQOL. 

#### 2.3.5. Cognitive Debriefing

This prefinal version was then tested and discussed with five lymphedema inpatients. 

#### 2.3.6. Review of Cognitive Debriefing Results and Finalisation

This was performed by the project manager.

#### 2.3.7. Proof Reading and Editing

The final version was then proofread by all translators involved in the forward translation and a doctoral candidate. Final editing and layout were performed by a professional designer with the instruction to produce a graphically simple layout to facilitate printing in everyday clinical use.

### 2.4. Study Population

Patients affected by lymphedema were recruited for interviews (1) when presenting at Leipzig University Hospital, Department of Plastic and Reconstructive Surgery, (2) in participating angiologist practices, (3) in physiotherapeutic practices and specialised health care supply stores, if their medical prescription included the diagnosis lymphedema, and (4) at meetings of patient support groups. To enable the enrolment of participants with reduced mobility and from multiple German-speaking countries (Germany, Austria, Switzerland), we also offered (5) online participation via patient support groups. Informed consent was obtained from every participant.

### 2.5. Statistics

Data were analysed using the software IBM SPSS Statistics, Version 29 (IBM Corporation, Armonk, NY, USA). Descriptive statistics were performed to characterise the epidemiologic characteristics of the sample by mean, mode, median of the standard deviation, and the standard error. To evaluate the German version of the LYMQOL Leg and prove its validity, different statistical procedures were performed addressing face, content, and construct validity. Additionally, reliability was examined by conducting test-retest and internal consistency analyses.

For comparison between LYMQOL Leg and SF-36 questionnaire, Pearson’s correlation was used. To compare the measurements of both questionnaires on both occasions the intra-class correlation was used as a two-way random effects model. Missing data were handled by multiple imputations using five imputed data sets. The level of significance was set at <0.05 for two-tailed tests.

### 2.6. Validation

#### 2.6.1. Content Validity

The extent to which the LYMQOL questionnaire was subjectively viewed as covering the concept it was intended to measure was evaluated by means of a questionnaire given to the participants on the first occasion of completing the LYMQOL. The questionnaire consisted of six questions, and the options for answers were dichotomised or open.

#### 2.6.2. Criterion and Construct Validity

To prove the construct validity, the LYMQOL scores were compared with the SF-36 MCS and PCS scores as ‘gold standards’ and with the patient’s reported perceived degree of swelling of the limbs. We hypothesized that the MCS correlated with the LYMQOL domains of appearance and mood. Further, we stated that the PCS correlated with the LYMQOL domains of function and symptoms. Thresholds for the correlation coefficient values were set up to be >0.50 (moderate to strong).

### 2.7. Test-Retest Reliability

Participants were asked to complete the German LYMQOL Leg and SF-36 twice with an interval of one week. Only data sets with both questionnaires completed twice were included in the analysis. The association between the two interviews was assessed with intra-class correlation coefficient (ICC). An ICC above 0.70 was considered acceptable. The standardised error of measurement (SEM) specifies the value of the error between the measurements and the smallest real difference (SRD) represents the smallest difference that is statistically significant. 

#### Internal Consistency

Cronbach’s alpha and factor analysis was performed to prove internal consistency. Value between 0.70–0.95 was set to be acceptable. In order to represent a reliable model, a total variance in the model for factor analyses of at least 50% was considered acceptable [45]. The associations between the patient’s perceived degree of limb lymphedema and the LYMQOL score for the domains were analysed with Kruskal–Wallis test and Spearman’s rank correlation coefficient.

## 3. Results

### 3.1. Translation Process

The translation process is described in detail in the methods section. Only a few minor differences were perceived between the seven forward translations. This might be attributed to the favourably simple language used in the original version. There was strong consensus on the usefulness of the added indicators and questions. A review of the backward translation showed near congruence to the original English version. The formatted final version of *LYMQOL Bein* is provided in the Supplementary Files.

### 3.2. Participants

One hundred and three persons with LLL were interviewed (Table 1). The LYMQOL Leg and SF-36 questionnaires were completed on both occasions. The participant’s average age was 53 years. The largest proportion of patients were women (92%). Patients were diagnosed with lymphedema on average 20 years ago. The mean BMI was 33 kg/m^2^. The largest proportion of respondents was employed (56), followed by pensioners (32). Two-thirds of participants suffered from stage 2 lymphedema. In 50% of cases, the lymphedema primarily affected the left leg. The answers indicated most frequently in LYMQOL Leg, and the calculated subscales of SF36 are displayed in Table 2.

#### 3.2.1. Completeness

Only participants completing LYMQOL Leg as well as SF-36 on both occasions were included. Among these, high rates of completeness were reached for both questionnaires. During the first interview round 19 (0.64%) of items in LYMQOL Leg remained unanswered, rising to 28 (0.94%) in the second interview round. The number of missing cells was slightly lower for SF-36 with 16 (0.56%) unanswered items in interview round one and 20 (0.70%) in round two.

#### 3.2.2. Face and Content Validity

Table 3 presents the face and content validity analysis for the German LYMQOL Leg. More than 90% of participants perceived the questions as easy to answer and the number of questions as appropriate and clear. In total, 77% of participants confirmed that no relevant aspects of life are not covered by the questionnaire. This left 18% who found that important areas of their life influenced by lymphedema were not covered in the questionnaire, namely vacations, sports, partnerships, family, friendships, and sexuality. Around 90% deemed no question unnecessary and almost 80% of participants had no further comments.

#### 3.2.3. Construct Validity

The analysis revealed a high correlation between the SF 36 subscale PCS and LYMQOL Leg domains of function and symptoms in both interviews, with Pearson’s correlation coefficients ranging from −0.6 to −0.83 (Table 4). With coefficients between −0.41 and −0.43, the appearance domain showed no correlation to the MCS subscale. In contrast, the LYMQOL Leg mood domain presented a high correlation with the MCS subscale (−0.69/−0.74). All tests showed a probability of error of ≤0.001.

The patient-reported lymphedema stages did not consistently correlate with the LYMQOL domain scores (Table 5). Only the domain of function demonstrated a significant difference between the lymphedema stages using a Kruskal–Wallis test. The correlation according to Spearman demonstrated no relation between the function domain and the lymphedema stage, demonstrated by a coefficient of 0.359 (*p* < 0.001). All other subgroups showed neither a significant difference between groups and clinical stage nor a significant correlation. However, the distribution of the lymphedema stages was unbalanced, with only 9 persons classified as lymphedema stage I, 60 as stage II, and 34 as stage III.

#### 3.2.4. Test–Retest Reliability

To explore reliability, differences in the completed answers between the first and second interviews were examined for both questionnaires. The consistency was classified as good for the domain of symptoms (ICC 0.68) and very good for every other subscale of the LYMQOL Leg (ICC 0.80–0.92) (Table 6).

#### 3.2.5. Internal Consistency

The calculation of the internal consistency of the German LYMQOL Leg version revealed Cronbach’s alpha values ranging from 0.76 to 0.89 (Table 7). In accordance with internationally accepted thresholds, these results were interpreted as very good [46]. 

In a further step, a factor analysis was performed. Every item demonstrated loading above 0.3. The cumulative explained variance was 52.7% for LYMQOL Leg with four components (Table 8). Therefore, the factor structure in the four domains was acceptable.

#### 3.2.6. Floor and Ceiling Effects

No floor effects were shown for the first interview. One person reached the lowest score in the domain of symptoms in the second interview (0.9%). Ceiling effects were observed in 2.9% of the participants in the function domain during the first interview. In total, 2.9% to 3.8% of the participants reached the highest score in all domains during the second interview. In conclusion, most of the participants had either a floor or ceiling score in the LYMQOL Leg domains (Table 9). 

## 4. Discussion

Within the scope of this study, we were able to translate, cross-culturally adapt, and validate the English LYMQOL Leg into German. The German version of the LYMQOL Leg met the quality criteria for measurement properties in the validation of health status questionnaires according to Terwee et al. concerning content validity, internal consistency, construct validity, agreement, reliability, floor or ceiling effects, and interpretability [47]. 

With FLQA-L being available in long and short forms, the question if an additional HRQoL PROM for lymphedema patients in German is necessary might be raised. Two key aspects justified initiating the process of translation and cross-cultural adaptation: awareness of FLQA-L’s existence seems to be rather low among practitioners and scientists. In fact, in the *German-Speaking Society for Microsurgery*’s 2019 Consensus on lympho-reconstructive microsurgery for secondary lymphedema, it was stated that translation and validation Lymph-ICF-LL is undertaken, not acknowledging the existence of FLQA-L [48]. The German 2017 guidelines on the diagnosis and treatment of lymphedema are currently under revision since they were valid only until 2022. Although compiled more than a decade after the introduction of FLQA-L, it is not mentioned. Instead, the use of EORTC-BR 23 is recommended for lymphedema of the arm and breast, and no option for lower limbs is designated [49]. More importantly, according to a systematic review of PROM for lymphedema based on the COSMIN framework, LYMQOL is clearly recommended over other PROMs, including FLQA-L and FLQA-LS [24]. The COSMIN methodology rates PROM on seven properties. FLQA-L was attributed with reliability, construct validity, and responsiveness but with low to very low quality of evidence, respectively. There was no sufficient evidence for its content validity, structural validity, internal consistency, and measurement error, partly due to insufficient reporting. FLQA-LS received a positive rating solely for its construct validity; the other six properties were not reported or indeterminate. LYMQOL Leg, on the other hand, showed content validity, structural validity, internal consistency reliability, and construct validity, meeting five out of seven criteria with moderate to high evidence. Although LYMQOL Leg did meet COSMIN methodology criteria for good evidence in PROM to their full extent, it must be noted that no other lymphedema PROM met all criteria, and LYMQOL-leg effectively was the only PROM to be “recommended with confidence” [24,50].

As for the Swedish and Dutch translations of LYMQOL, the construct validity was assessed by comparing the LYMQOL domains of function and symptoms with the SF-36 subscale PCS and the LYMQOL domains of appearance and mood with the SF-36 subscale MCS. The leg version demonstrated statistically significant correlations in three of four domains with PCS and MCS. However, the LYMQOL domain of appearance demonstrated no significant correlation with the SF-36 subscale MCS. These findings align with the results of van de Pas et al. and Borman et al. in their respective validations after translation [31,35]. This leads us to assume a general rather low construct validity of LYMQOL regarding mental aspects.

We found significant correlation and intraclass correlation coefficients in our German LYMQOL version, as did Keeley et al. in their original publication [25]. In order to establish construct validity, we analysed the correlation between the clinical lymphedema stage and the LYMQOL outcome but were unable to find a significant correlation. Similarly, Keeley et al. compared limb volume with LYMQOL scores with no significant correlations [25]. In contrast, other PROM for lymphedema patients were able to prove a correlation between objective limb volume and HRQoL, among them the Swedish LYMQOL Leg [33,51]. The interference of comparatively high weight and BMI measured in our cohort might be a contributing factor to these differing results. 

The test–retest correlation analyses revealed a high degree of consensus, with correlation coefficients around 0.90 (ICC), with the exception of the domain of symptoms. The internal consistency was good to very good. Other studies obtained similar results [32,33,35,38,39].

The original publication and following translations demonstrated that lymphedema patients sensed the instrument was easy to use [25,33]. Nevertheless, 18% of participants felt that certain aspects of their lives influenced by lymphedema were not represented in the questionnaire (vacations, sports, partnerships, family, friendships, and sexuality). Adding these aspects as distinct items was already denied by Keeley et al. [25]. In our opinion, examining these perceived omissions does not entail a need for increasing the number of questions. The English original’s allure lies in the broad simplicity of its questions, thus actually covering the indicated missing aspects as follows: Vacations and sports fall into the category of leisure activities covered by question 2, including a text field for the patient’s examples. Family and friendships are covered by the question “Does it affect your relationship with other people?”, and sexuality can be inferred from the question “Does it affect your relationship with your partner?”.

The final version of the German LYMQOL slightly differs from the original English version by Keeley et al. During the revision process, Keeley et al. removed items if deemed redundant after further statistical analysis [25]. Among others, the question “Does it affect your relationship with your partner?” was removed from the final questionnaire due to poor correlation and since it was thought to be sufficiently answered by the question “Does it affect your relationship with other people?”. After careful consideration, we argue that explicitly differentiating partnership and relationship with others better reproduces a deepened sense of intimacy, including a sexual relationship. After our own interviews, we see this decision accredited by patients explicitly stating they did not perceive their sexuality to be sufficiently covered by the questionnaire. Furthermore, it did not seem comprehensible why patients with ULL were asked “Do you feel tired?” in LYMQOL Arm and patients with LLL in LYMQOL Leg were not. We thus decided to adopt this question into LYMQOL Leg. Consequently, our proposed German version of LYMQOL consists of 31 items in total instead of the original 27, including the question on affected limbs and perceived severity of leg swelling. The number of items is identical in the function and mood domains and increased in the appearance (eight versus seven) and symptoms (six versus five) domains. Since the score is calculated by dividing by the number of questions, our increased item number does not affect scoring.

We also included minor alterations after putting the questionnaire to the test. During cognitive debriefing and the interviews, whether in person or online, participants were always asked for remarks on the questions. For the main items, checkboxes are included to indicate answers on the Likert-like scale. The original publication states that patients should write *N/A* in the boxes if items are not applicable. After several interviews, we observed the patient’s irritation by this, consequently writing explanations or not answering questions at all. Therefore, we added a fifth column next to the four-grade Likert-like scale for *not applicable* in order to enhance clarity. Multiple patients emphasised that not only the swelling of the leg affected them but that the treatments were particularly restricting in multiple aspects of their well-being. Every participant in this interview underwent conservative therapy, including manual decongestive therapy and wearing medical-grade compression garments. Understandably, these therapy methods might be perceived as inconvenient, time-consuming, financially challenging, disfiguring, stigmatising, and even painful and thus deteriorate HRQoL. In order to adequately accommodate this from the patient’s perspective important factor, we decided to reword the main question by adding “How much do your swollen leg *and its therapy* affect […]?” To increase the examiner’s comfort, we added a table for recording the domain scores and results. It also includes the number of items per domain, which is required to calculate the score; previously, this had to be counted manually. With the help of a professional graphic designer, we clearly outlined each domain while keeping the overall design minimalistic and printer friendly for routine use. However, the further modifications decided upon by our group do not influence the actual score.

During the translations, we ensured having equal numbers of female and male physicians and professional translators partake in the process to account for possible gender-specific differences in the use of language, especially in a questionnaire involving body function, appearance, and mood [52].

While 103 patients with LLL is among the higher study population numbers in the series of LYMQOL translations, the low number of participants with stage I lymphedema is a limiting factor to our version’s applicability in this specific cohort. Another limitation is the unequal distribution of sexes among participants. However, the surplus of female participants in our study population does align with LLL mostly affecting patients with gynaecological cancers [5]. Women seeking professional help in medical care and participating in patient support groups more often than men might also be causative factors for our female-dominated patient recruiting [53]. Further limitations are the unrepresented body parts like the feet, hands, or the genital area, as also mentioned by Keeley et al. [25,33]. A source of uncertainty in our data collection is the inclusion of mere online interviews, as this did not allow for securing the diagnosis of lymphedema by a physician. We explicitly stated that the interviews are aimed at lymphedema, not lipedema patients when contacting patient support groups since many groups welcomed both patient groups. As another countermeasure, patients filling in the forms online were asked to check their doctor’s prescriptions for diagnosis and stage of lymphedema.

We plan to re-evaluate the questionnaire based on further comments on the ongoing online validation process as well as long-term clinical use, e.g., in longitudinal studies in patients seeking surgical treatment pre- and postoperatively.

A translated and validated German version of LYMQOL for ULL is still lacking. 

## 5. Conclusions

We translated and cross-culturally adapted the English LYMQOL Leg questionnaire to German. The validity of the resulting *LYMQOL Bein* questionnaire was demonstrated; thus, it is now available to healthcare providers for the evaluation of HRQoL in patients with lower limb lymphedema. 

## Figures and Tables

**Table 1:** Descriptive data of the study population interviewed for translation and validation of the German version of LYMQOL Leg.

	**LYMQOL Leg**
Number of participants	103
Age	53 ± 13 (26–85) years
Sex	8 m/95 f (8% m/92% f)
Time between diagnosis and interview	21 ± 6 (4–40) years
Body weight	94 ± 25 (51–180) kg
Height	167 ± 7 (153–188) cm
BMI	33 ± 9 (18–72) kg/m^2^
Employment	56 employed/8 freelancer/32 pensioned/3 unemployed/4 missing
Graduation	68 secondary school/17 high school/16 universitiy/2 missing
Smoking	11 smokers/66 non-smokers/25 ex-smokers/1 missing
**ISL Lymphedema Stage**	
Stage I	9
Stage II	60
Stage III	34
**Which body area is affected by lymphedema?**	
Left leg	52/50%
Right leg	12/12%
Bilateral	39/38%

**Table 2:** Most frequent scores in LYMQOL Leg and SF-36 in both interviews.

	**LYMQOL Leg**	
	**First Interview** **(Modus/Median)**	**Second Interview** **(Modus/Median)**
1. question	3/4	3/3
2. question	4/3	3/3
Function	2/3	2/3
Appearance	2/3	2/3
Symptoms	2/3	2/2
Mood	2/2	2/2
LYMQOL score	5/5	5/5
	**SF 36**	
Physical function (PF)	50.3 ± 27.9 (0–100)	48.9 ± 27.7 (0–100)
Role physical (RP)	36.1 ± 40.1 (0–100)	37.1 ± 38.9 (0–100)
Role emotional (RE)	53.2 ± 43.6 (0–100)	52.1 ± 44.9 (0–100)
Vitality (VT)	37.1 ± 20.8 (0–95)	35.4 ± 21.4 (0–100)
Mental health (MH)	56.0 ± 20.5 (0–100)	55.5 ± 21.9 (4–100)
Social functioning (SF)	58.9 ± 25.2 (0–100)	57.6 ± 25.0 (0–100)
Bodily pain (BP)	49.3 ± 28.1 (0–100)	46.4 ± 27.5 (0–100)
General health (GH)	39.7 ± 22.1 (5–95)	39.4 ± 24.4 (0–95)
Physical Component Summary (PCS)	50.0 ± 10.8 (29.8–70.8)	50.0 ± 10.8 (29.8–70.8)
Mental Component Summary (MCS)	49.9 ± 10.4 (29.3–74.4)	50.0 ± 10.5 (25.2–74.8)

**Table 3:** Face and content validity of the German LYMQOL Leg version presented as absolute value/relative value.

		Yes	No	No Answer
1.	Was the questionnaire easy to answer?	95/92%	5/5%	3/3%
2.	Was the number of questions appropriate?	93/90%	6/6%	4/4%
3.	Were the questions clear?	93/90%	7/7%	3/3%
4.	Is there an important area of life in which lymphedema impacts your quality of life that is not included in the questionnaire?	19/18%	79/77%	5/5%
5.	Was a question unnecessary?	2/2%	94/91%	7/7%
6.	Do you have any comments about this questionnaire?	18/17%	82/80%	3/3%

**Table 4:** Correlations between LYMQOL Leg and the SF 36 questionnaire.

SF-36	PCS				MCS			
	First Interview		Second Interview		First Interview		Second Interview	
LYMQOL	r_s_	*p*	r_s_	*p*	r_s_	*p*	r_s_	*p*
Function	−0.81	≤0.001	−0.83	≤0.001				
Symptoms	−0.63	≤0.001	−0.60	≤0.001				
Appearance					−0.43	≤0.001	−0.41	≤0.001
Mood					−0.69	≤0.001	−0.74	≤0.001

**Table 5:** Correlation between LYMQOL Leg score and ISL lymphedema stage.

	I	II	III	Kruskal–Wallis Test	r_s_	*p*
Number of participants	9	60	34	*p*-value		
Function	2.1 (1.5–3.1)	2.5 (1.0–3.8)	2.9 (1.5–4.1)	0.001	0.359	<0.001
Appearance	2.4 (1.3–3.5)	2.7 (1.1–4.0)	2.8 (1.1–3.8)	0.112	0.205	0.38
Symptoms	1.8 (1.2–3.0)	1.9 (0.7–3.0)	1.9 (0.8–2.8)	0.511	0.045	0.655
Mood	2.2 (1.1–3.0)	1.9 (1.0–3.1)	1.8 (0.7–3.3)	0.319	−0.139	0.162
Global Score	2.1 (3.0–7.0)	5.0 (1–9)	4.1 (0–10)	0.168	−0.180	0.07

r_s_ = Spearman’s rank correlation coefficient.

**Table 6:** Test–retest reliability of German LYMQOL Leg.

	First Interview	Second Interview					
	Mean Score ± Standard Deviation	Mean Score ± Standard Deviation	ICC	*p*	Difference Means	SEM	SRD
Function	2.6 ± 0.7	2.5 ± 0.7	0.92	≤0.001	0.1	0.07	0.19
Appearance	2.7 ± 0.7	2.7 ± 0.7	0.89	≤0.001	0.0	0.06	0.16
Symptoms	1.9 ± 0.5	2.4 ± 0.7	0.68	≤0.001	−0.5	0.07	0.19
Mood	1.9 ± 0.6	2.3 ± 0.8	0.80	≤0.001	−0.4	0.07	0.19
Global score	4.6 ± 2.3	4.7 ± 2.3	0.90	≤0.001	−0.1	0.24	0.66

**Table 7:** Cronbach’s alpha values for German LYMQOL Leg.

	Interview 1		Interview 2
	Number of Items	Cronbach’s Alpha Coefficient	Cronbach’s Alpha Coefficient
Function	8	0.89	0.90
Appearance	8	0.76	0.77
Symptoms	6	0.79	0.83
Mood	6	0.89	0.87

**Table 8:** Factor analysis of the German LYMQOL Leg.

	Question Number	Component			
		1	2	3	4
Function	1	**0.677**	0.122	0.208	0.073
	2	**0.701**	0.063	0.138	0.120
	3	**0.662**	0.096	0.214	−0.015
	4	**0.861**	0.104	0.188	−0.038
	5	**0.618**	−0.132	−0.028	0.128
	6	**0.801**	0.114	0.020	0.162
	7	**0.656**	0.055	0.068	0.278
	8	**0.681**	0.078	0.031	0.119
Appearance	9	**0.308**	**0.359**	**0.523**	−0.017
	10	0.195	0.094	**0.835**	0.059
	11	0.144	0.216	**0.762**	0.109
	12	0.034	0.074	0.020	**0.628**
	13	0.172	0.062	**0.410**	**0.526**
	14	0.136	**0.491**	**0.543**	0.119
	15	0.263	0.153	0.005	**0.592**
	16	**0.436**	**0.377**	0.133	**0.365**
Symptoms	17	**0.432**	0.270	**0.326**	−0.008
	18	**0.468**	**0.336**	0.082	−0.011
	19	**0.391**	0.245	0.160	0.015
	20	**0.396**	**0.375**	0.115	0.175
	21	**0.501**	0.252	0.263	0.161
	22	**0.525**	**0.376**	0.275	0.295
Mood	23	0.231	**0.558**	0.050	0.054
	24	0.060	**0.815**	0.079	0.068
	25	0.075	**0.772**	0.167	0.031
	26	0.002	**0.742**	**0.310**	0.079
	27	0.188	**0.832**	0.156	0.107
	28	0.095	**0.821**	0.081	0.139

Cumulative explained variance: 52.7%; Bold numbers are factors loading > 0.3.

**Table 9:** Floor and ceiling effects. Participants had the lowest (floor) and highest (ceiling) scores in the LYMQOL Leg domains in the two interviews.

	First Interview	Second Interview	First Interview	Second Interview
	Lowest Score (=0, Floor) % (n/N)	Lowest Score (=0, Floor) % (n/N)	Highest Score (=4, Ceiling) % (n/N)	Highest Score (=4, Ceiling) % (n/N)
Function	0% (0/103)	0% (0/103)	2.9% (3/103)	3.8% (4/103)
Appearance	0% (0/103)	0% (0/103)	0.9% (1/103)	2.9% (3/103)
Symptoms	0% (0/103)	0.9% (1/103)	0% (0/103)	3.8% (4/103)
Mood	0% (0/103)	0% (0/103)	0% (0/103)	2.9% (3/103)

## Data Availability

The data presented in this study are available in the publicly accessible repository *Zenodo* at https://zenodo.org/records/10462670 (accessed on 1 February 2024).

## Supplementary Materials

The following supporting information can be downloaded at: https://www.mdpi.com/article/10.3390/healthcare12030409/s1. Translated, cross-culturally adapted and validated German version of LYMQOL Leg by Keeley et al., termed LYMQOL Bein by Nuwayhid et al.
