# Supplementary material for: Translation, Cross-Cultural Adaptation and Validation of the Lymphedema Quality of Life Questionnaire (LYMQOL) in German-Speaking Patients with Lymphedema of the Lower Limbs
Source: Healthcare (Basel). 2024 Feb 5;12(3):409. doi: 10.3390/healthcare12030409 (PMC10855540; doi:10.3390/healthcare12030409)
Supplement: Supplementary file 1 [file healthcare-12-00409-s001.zip › healthcare-2836244-supplementary.pdf]

Fragebogen zur Erfassung der Lebensqualität bei Lymphödem des Beines

LYMQOL Bein

Sehr geehrte Patient*innen,
der vorliegende Fragebogen dient der Erfassung des Einflusses des bei Ihnen bestehenden Lymphödems auf Ihre Lebensqualität. Bitte beziehen Sie dabei alle Fragen auf die Schwellung selbst, die notwendigen Behandlungen (bspw. manuelle Lymphdrainage, Kompressionskleidung) sowie eventuell auftretende Komplikationen (bspw. Erysipel/Wundrose). Vielen Dank!

Alternativ: Patient*innenetikett

Name: ____________________

Geburtsdatum: ____________________

	Datum: ____________________	**gar nicht** (1)	**etwas** (2)	**recht stark** (3)	**sehr stark** (4)	**n. z.** nicht zu-treffend
	**1. Als wie stark empfinden Sie die Schwellung des linken Beines?**	☐	☐	☐	☐	☐
	**2. Als wie stark empfinden Sie die Schwellung des rechten Beines?**	☐	☐	☐	☐	☐
FUNKTION	**3. Wie stark beeinträchtigen das geschwollene Bein und seine Behandlung Sie bei folgenden Alltagsaktivitäten?**					
	a. Gehen	☐	☐	☐	☐	☐
	b. Vornüberbeugen, z.B. beim Schuhe anziehen oder der Fußpflege	☐	☐	☐	☐	☐
	c. Stehen	☐	☐	☐	☐	☐
	d. Aus dem Sitzen aufstehen	☐	☐	☐	☐	☐
	e. Im Beruf	☐	☐	☐	☐	☐
	f. Bei der Hausarbeit	☐	☐	☐	☐	☐
	**4. Wie stark sind Ihre Freizeitaktivitäten / Ihr Sozialleben dadurch beeinträchtigt?**	☐	☐	☐	☐	☐
	Bitte nennen Sie Beispiele:					
	**5. Wie sehr sind Sie auf Hilfe angewiesen?**	☐	☐	☐	☐	☐
KÖRPERBILD	**6. Wie sehr denken Sie, verändert die Schwellung Ihr Aussehen?**	☐	☐	☐	☐	☐
	**7. Wie schwierig ist es, Kleidung zu finden, die Ihnen passt?**	☐	☐	☐	☐	☐
	**8. Wie schwierig ist es, Kleidung zu finden, die Sie gerne tragen?**	☐	☐	☐	☐	☐
	**9. Wie schwierig ist es, passende Schuhe zu finden?**	☐	☐	☐	☐	☐
	**10. Wie schwierig ist es, passende Socken / Strümpfe / Strumpfhosen zu finden?**	☐	☐	☐	☐	☐
	**11. Beeinflusst die Schwellung Ihr Selbstbild?**	☐	☐	☐	☐	☐
	**12. Beeinflusst die Schwellung Ihre Partnerschaft?**	☐	☐	☐	☐	☐
	**13. Beeinflusst die Schwellung Ihre Beziehungen zu anderen Menschen?**	☐	☐	☐	☐	☐

BITTE WENDEN → Fortsetzung auf der Rückseite

		gar nicht (1)	etwas (2)	recht stark (3)	sehr stark (4)	n. z. nicht zutreffend
SYMPTOME	**14. Haben Sie Schmerzen durch das Lymphödem?**	☐	☐	☐	☐	☐
	**15. Verspüren Sie ein Taubheitsgefühl im geschwollenen Bein?**	☐	☐	☐	☐	☐
	**16. Verspüren Sie ein Kribbeln oder „Ameisenlaufen" im geschwollenen Bein?**	☐	☐	☐	☐	☐
	**17. Fühlt sich das geschwollene Bein schwach an?**	☐	☐	☐	☐	☐
	**18. Fühlt sich das geschwollene Bein schwer an?**	☐	☐	☐	☐	☐
	**19. Fühlen Sie sich müde oder erschöpft?**	☐	☐	☐	☐	☐
STIMMUNG	**Hatten Sie in der vergangenen Woche…**					
	**20. Probleme mit Schlaf?**	☐	☐	☐	☐	☐
	**21. Konzentrationsschwierigkeiten, z.B. beim Lesen?**	☐	☐	☐	☐	☐
	**22. Gefühle der Anspannung?**	☐	☐	☐	☐	☐
	**23. Sorgen?**	☐	☐	☐	☐	☐
	**24. Erhöhte Reizbarkeit?**	☐	☐	☐	☐	☐
	**25. Niedergeschlagenheit?**	☐	☐	☐	☐	☐

26. Wie würden Sie Ihre Lebensqualität derzeit insgesamt bewerten?
Bitte vergeben Sie einen Punktwert auf der folgenden Skala:

☐	☐	☐	☐	☐	☐	☐	☐	☐	☐	☐
**0**	**1**	**2**	**3**	**4**	**5**	**6**	**7**	**8**	**9**	**10**

schlecht → hervorragend

Vielen Dank für Ihre Teilnahme!

Der vorliegende Fragebogen ist eine adaptierte Übersetzung des englischen LYMQOL Leg, welcher von Prof. Vaughan Keeley und Kollegen etabliert wurde (Keeley V, Crooks S, Locke J, et al. A quality of life measure for limb lymphoedema (LYMQOL). Journal of Lymphoedema 2010: pp. 26–37). Die Methodik zu Übersetzung, Adaptation und Validierung wurde veröffentlicht unter Nuwayhid R et al.: Translation, Cross-Cultural Adaptation and Validation of the Lymphedema Quality of Life Questionnaire (LYMQOL) in German-Speaking Patients with Lymphedema of the Lower Limbs. Healthcare. 2024; 12(3):409. https://doi.org/10.3390/healthcare12030409

Bei Fragen oder Anmerkungen zu diesem Fragebogen wenden Sie sich bitte an:
Dr. med. Rima Nuwayhid
Klinik für Orthopädie, Unfallchirurgie und Plastische Chirurgie
Universitätsklinikum Leipzig AöR
Liebigstraße 20
04103 Leipzig

Für die Untersuchenden: Auswertung
Pro Domäne werden die den Antworten zugeordneten Zahlenwerte addiert und durch die Anzahl der Fragen pro Domäne dividiert (Funktion ÷ 8, Körperbild ÷ 8, Symptome ÷ 6, Stimmung ÷ 6). Hierdurch ergeben sich Werte von 0 bis 4, wobei höhere Werte eine stärkere Einschränkung der Lebensqualität widerspiegeln. Sind die Hälfte oder mehr Fragen einer Domäne nicht beantwortet oder als nicht zutreffend gekennzeichnet, kann diese Domäne nicht ausgewertet werden.

	Funktion (÷ 8)	Körperbild (÷ 8)	Symptome (÷ 6)	Stimmung (÷ 6)
**ERGEBNIS**				
